# The evolution of nonlinear mixed effects modeling in pharmacometrics: toward AI-based variational autoencoders

**DOI:** 10.1007/s10928-026-10044-9

**Published:** 2026-07-08

**Authors:** Jan Rohleff, Gilbert Koch, Johannes Schropp

**Affiliations:** 1 Department of Mathematics and Statistics, University of Konstanz, Konstanz, Germany; 2 Pediatric Pharmacology and Pharmacometrics, University Children’s Hospital Basel (UKBB), University of Basel, Basel, Switzerland

**Keywords:** Nonlinear Mixed Effects Modeling, First-Order Conditional Estimation, Stochastic Approximation Expectation Maximization, Variational Autoencoder, Artificial Intelligence, Machine Learning

## Abstract

The evolution of nonlinear mixed effects (NLME) modeling reflects a continuous cycle of innovation based on advances in numerical methods and computational power. This commentary outlines the evolution of NLME modeling that began with linearization-based approaches in the 1980s, progressed through sampling-based methods in the 2000s, and is now entering a new phase shaped by AI. Variational autoencoders bridge classical NLME modeling with AI-based methods allowing the development and application of AI-augmented PMX models. This opens the route for integrating multimodal data and addressing increasingly complex modeling challenges.

## Introduction

Nonlinear mixed effects (NLME) modeling is a key tool in pharmacometrics (PMX). The evolution of NLME modeling is a continuous cycle of innovation, where breakthroughs in computational statistics and applied mathematics have been rapidly adapted to PMX. Core of this evolution is the maximization of the marginalized likelihood

1 $$\mathcal{L}\left(y;\theta \right)=\int p\left(y,\phi ;\theta \right) d\phi =\int p\left(y|\phi ;\theta \right) p\left(\phi ;\theta \right) d\phi$$

in the NLME framework, where *y* denotes the observed data, *ϕ* the individual parameters, and *θ* the population parameters. The integration in Eq. (1) is performed over the individual parameters *ϕ*, which in many NLME formulations is equivalently formulated as integration over the random effects *η*. Parameter estimation in NLME modeling requires frequent evaluation of Eq. (1). However, this involves integration over individual parameters, which in general cannot be performed analytically.

## Historical overview of computational methods

The evolution of NLME modeling has closely followed advances in numerical methods and computational power. As a result, each generation has adopted the most advanced methodologies available at its time to maximize the marginalized likelihood $\mathcal{L}$ in Eq. (1). In this section, we briefly review this evolution from its origins in the 1980s to the current state of the art.

### The 1980s - linearization-based methods

NLME modeling became feasible in the early 1980s, driven by advances in likelihood approximation methods, increasing computational power, and improved numerical techniques for ordinary differential equations (ODEs), sensitivity analysis, and optimization.

Early approaches approximated the likelihood $\mathcal{L}$ in Eq. (1) by linearizing it with respect to the individual parameters *ϕ* (centered at zero expectation) and maximizing it using gradient-based optimization. This design principle underlies several widely used algorithms, such as First-Order (FO), First-Order Conditional Estimation (FOCE), and Laplace approximation [1].

These techniques were first implemented in NONMEM [2]. However, while computationally efficient, linearization-based methods can be biased [3], when applied to nonlinear models or in presence of large inter-individual variability.

### The 2000s - sampling-based methods

NLME modeling further evolved with advances in sampling-based methods rooted in Bayesian statistics in the early 2000s. Iterative optimization algorithms, such as Expectation-Maximization (EM) (1977) [4], combined with sampling techniques like Markov Chain Monte Carlo (MCMC, 1950s) and Metropolis–Hastings (1970s) provided the theoretical foundation. These methods became practically applicable as computational power increased.

Leveraging these advances, the stochastic approximation expectation maximization (SAEM) [5, 6], a sampling-based method to approximate the likelihood $\mathcal{L}$ was introduced. SAEM iteratively samples the posterior random effects using MCMC in the expectation (E)-step and updates population parameters in the maximization (M)-step, and does not need any gradients. This provides a more accurate approximation of the likelihood $\mathcal{L}$ and, under standard regularity conditions, it converges to a local maximum of $\mathcal{L}$ [6].

The algorithm was first implemented in Monolix [7]. SAEM overcomes limitations of linearization-based methods. It provides more accurate estimates for nonlinear models, sparse data [8, 9], and better handles large inter-individual variability [5]. Additionally, its more global search strategy makes it less sensitive to initial parameter guesses, although it can be computationally demanding, particularly for large data sets, due to the required number of ODE evaluations.

### Summary

Classical methods such as FO, FOCE and SAEM are the current gold standard in NLME modeling. These techniques became the standard tools for analyzing complex PMX models. An overview of their development up to the early 2000s can be found in Mentré [10].

## The 2020s - AI-based methods for NLME modeling

The current decade marks a pivotal shift in PMX, driven by the rapid advancement of artificial intelligence (AI). These developments rely on new gradient-based optimization methods, including backpropagation, which enable efficient learning of complex neural network models [11]. AI-based approaches have gained increasing interest in PMX [12–15], due to their potential to improve automation, computational efficiency, and model flexibility.

### Early AI-NLME approaches

Early approaches integrated AI components into established NLME frameworks. These approaches, exemplified in [16], demonstrate the potential of combining AI with established NLME principles. At the same time, they reveal practical limitations of established NLME frameworks. Classical estimation algorithms have been developed and optimized for traditional NLME modeling, not for scalable AI-augmented neural network settings [17, 18]. Summarizing, these investigations suggest that combining NLME modeling with modern AI components is highly promising but introduces new requirements. This motivates the development of methods that better align NLME and AI approaches.

### Variational autoencoders in NLME modeling

The VAE framework can be introduced to NLME modeling in two steps. First, a standard formulation can be interpreted as an AI-based reformulation of stochastic approximation methods such as SAEM, in which the MCMC step is replaced by a recurrent neural network (RNN)-based sampling procedure. Second, an extension is introduced that enables a modular and flexible framework, allowing the integration of additional AI components and mathematical optimization methods into NLME modeling.

#### VAE-NLME standard formulation

The VAE architecture consists of two main components, encoder and decoder. The encoder, typically implemented using RNNs, provides a smooth approximation of the posterior distribution of the individual parameters *ϕ* (so-called variational inference), from which samples are drawn. Based on these samples, the decoder evaluates the PMX model and computes the model predictions.

An important advantage of this approach is that the VAE combines efficient gradient-based optimization with the accuracy and robustness of sampling-based MCMC methods.

The accuracy of population parameter estimates depends on how well the true posterior distribution is approximated by the neural networks [19]. Using a family of Gaussian distributions, log-likelihood values comparable to classical approaches can be achieved [20], while significantly reducing computation time [19]. A VAE formulation adapted to NLME is presented in [20], while related variational approaches can be found in [21–23].

#### Modular AI extensions of the VAE-NLME framework

Beyond reproducing classical solutions, another advantage of VAEs is their modular and fully learnable framework. In contrast to early approaches (Sect. "Early AI–NLME Approaches"), VAEs combine the advantages of classical methods, stochastic sampling and efficient gradient-based optimization, into a single unified learning framework. This enables joint learning of classical NLME parameters and additional AI components in an optimal manner, without numerical and structural limitations. Furthermore, the modular structure of VAEs allows the integration of additional mathematical concepts within the same framework.

Up to date, several VAE extensions have been proposed for solving PMX models. For example, a VAE combined with a mixed integer optimization solver in the update step of the population parameters enables automated and simultaneous estimation of population parameters and covariate selection within a single run [20]. In [24], Neural ODEs were incorporated into a VAE-NLME framework to automatically learn the dynamics of time-varying covariates. In [25], the entire structural model was defined by a Neural ODE, while a VAE was applied for NLME modeling.

The key properties and differences between FO/FOCE, SAEM, and VAE approaches for NLME modeling are summarized in Table 1.

**Table 1:** Comparison of NLME estimation methods: FO/FOCE, SAEM, and VAE

Feature	FO/FOCE	SAEM	VAE
**Core Characteristics**			
Decade Introduced	1980s	2000s	2020s
Core Principle	Linearized likelihood	Stochastic approximation EM	Variational inference (AI-based)
Optimization	Deterministic gradient	Stochastic optimization (gradient-free)	Stochastic gradient (Backpropagation)
Posterior	Deterministic conditional estimate	MCMC sampling	RNN-based sampling
Solution Quality	Local approximation (uncontrolled error)	Asymptotically exact (sufficient samples)	RNN posterior approximation (controllable error)
**Computational Efficiency**			
Individual Parameters	Subject-specific optimization	Subject-specific MCMC sampling	Shared RNN sampling
Effort	Grows with population size	Grows with population size	Scalable AI setting (fixed RNN weights)
Computational Cost (ODE Solver Calls)	Low to moderate	Moderate	Low to moderate
**Flexibility** **&** **AI Integration**			
Data Scope	Standard measurement data	Standard measurement data	Standard and multimodal data
AI Compatibility	Up to moderate nonlinearities	Up to moderate dimensions	Native AI framework
Current Role	Classical NLME	Classical NLME	NLME-AI bridge
**Trade-off Summary**			
Strength	Fast for simple models	Robust and asymptotically exact	Fast, robust, flexible and highly scalable
Limitation	Potential bias in nonlinear models	Computationally intensive	Accuracy depends on RNN posterior approximation

## The future - AI-augmented PMX models

The history of PMX has already demonstrated AI’s potential to automate and augment NLME modeling. With VAEs, a method is now available that bridges classical NLME modeling and AI, providing a framework in which the full potential of AI can be realized.

Consequently, AI-based methods can be integrated directly into NLME modeling wherever they are needed, allowing the development and application of AI-augmented PMX models. Beyond current achievements, we identify several areas where VAEs will fundamentally advance the field of PMX.

### Learning the structural model and unknown dynamics

A central task of classical PMX models is the need to predefine the structural model. VAEs combined with Neural ODEs suggest strong potential to move beyond predefined structural models towards data driven discovery of underlying system dynamics within the NLME framework. In [26], it is shown that structural model components can be recovered from data using low-dimensional Neural ODEs. Combining this idea with a VAE-based NLME framework capable of handling higher-dimensional Neural ODEs may enable the fully data-driven and automated learning of higher-dimensional structural models.

### Incorporating multimodal data

**Fig. 1:**
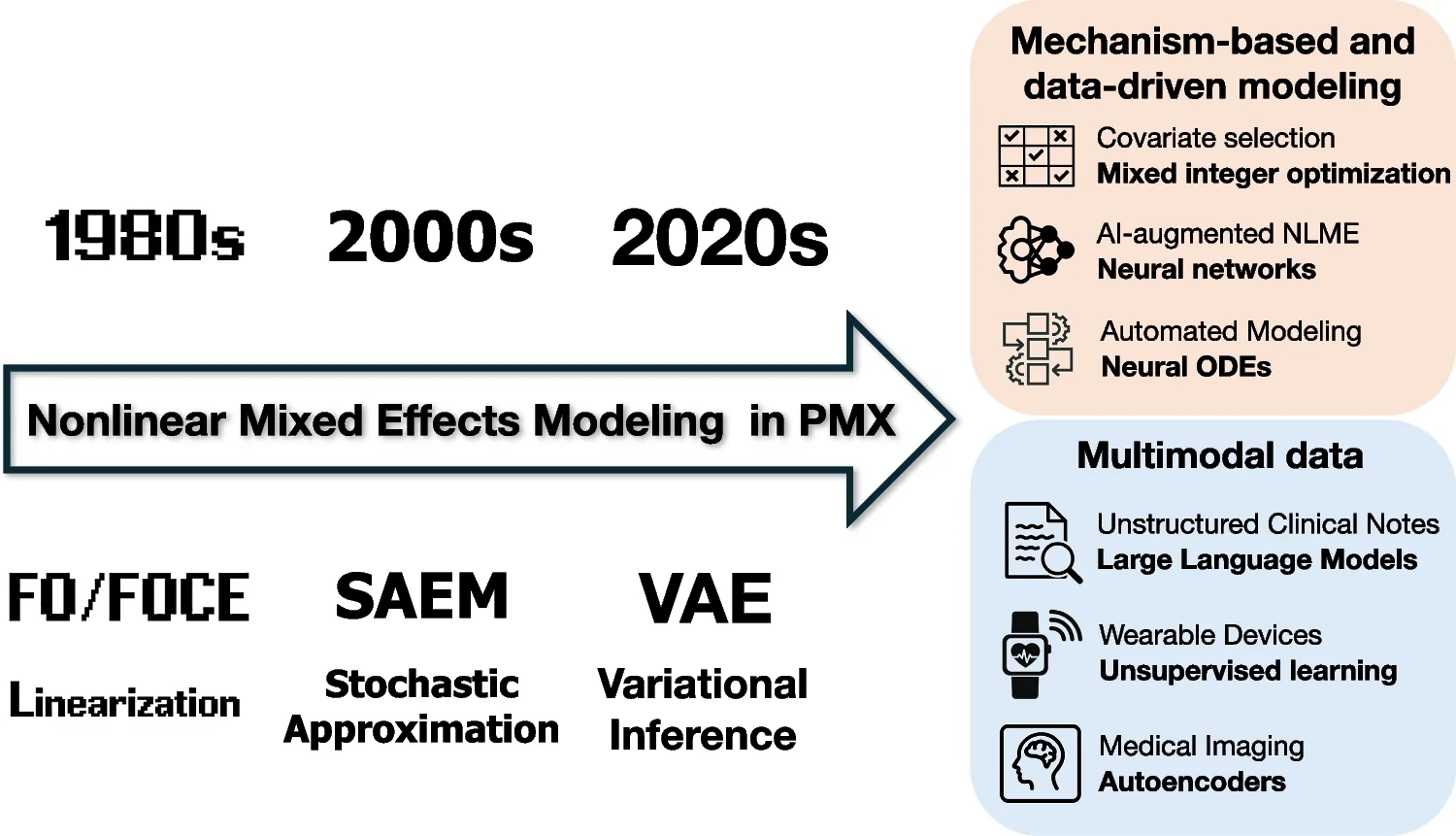
The evolution of NLME modeling to AI-augmented PMX

Patient data are typically far richer than what is commonly utilized in traditional PMX models. By leveraging VAEs for NLME modeling, multimodal data can be systematically integrated into PMX models, see Fig. 1. In the following, we present some data sources that on one hand increase PMX model complexity, but on the other hand will lead to AI-augmented PMX models.

Medical imaging plays an increasingly important role in modern healthcare due to its ability to non-invasively visualize anatomical structures and pathological changes. Recent advances in AI have significantly expanded the possibilities for automated image analysis. Hence, these developments open new opportunities to incorporate such high-dimensional information into AI-augmented PMX models.

Wearable devices have gained widespread adoption, e.g. as home monitoring tools, due to their increased accessibility and ability to continuously capture physiological data in real time. Recent advances have further enhanced the ability to analyze these data streams and transform them into digital biomarkers. Consequently, such data are a rich source of information beyond traditional clinical measurements, e.g. those performed at clinical visits.

Unstructured clinical notes represent a major component of electronic health records. Despite their richness, such data is difficult to analyze using conventional methods due to their heterogeneous and non-standardized nature. Recent advances in natural language processing have improved the ability to extract information from such free-form data sources and integrate them in PMX models [27].

Together, these developments highlight the potential of a VAE-based NLME framework to integrate diverse data sources and AI-based methods, paving the way towards more automated, multimodal data driven AI-augmented PMX models.

## Conclusion

The evolution of NLME modeling reflects a continuous cycle of innovation. Mathematical concepts first emerge in theory and are subsequently adapted to PMX. NLME modeling began with linearization-based approaches, progressed through sampling-based methods, and is now entering a new phase shaped by AI.

VAEs serve as the architectural bridge for this transition, as they enable the flexible integration of high-dimensional AI components into the classical NLME modeling. Consequently, PMX researchers can address PMX models of greater scale and complexity that were previously computationally too challenging.

Summarizing, the continuous cycle of theoretical advancement and practical implementation ensures that NLME modeling remains a dynamic and evolving field. With the emergence of new AI methodologies, innovations can be directly incorporated, further enriching classical NLME modeling.

## Data Availability

No datasets were generated or analysed during the current study.
